# Associations between diabetes and risk of short-term and long-term nursing home stays among older people receiving home care services: A nationwide registry study

**DOI:** 10.1186/s12877-024-05403-5

**Published:** 2024-10-09

**Authors:** Tonje Teigland, Jannicke Igland, Marit Graue, Kjersti M. Blytt, Johannes Haltbakk, Grethe S. Tell, Kåre I. Birkeland, Truls Østbye, Marit Kirkevold, Marjolein M. Iversen

**Affiliations:** 1 Department of Health and Caring Sciences, Western Norway University of Applied Sciences, Bergen, Norway; 2 Department of Global Public Health and Primary Care, University of Bergen, Bergen, Norway; 3 Institute of Clinical Medicine, University of Oslo, Oslo, Norway; 4 Department of Transplantation Medicine, Oslo University Hospital, Oslo, Norway; 5 Department of Family Medicine and Community Health, Duke University, Durham, NC USA; 6 Department of Nursing and Health Promotion, Oslo Metropolitan University, Oslo, Norway

**Keywords:** Diabetes, Home care services, Older people, Nursing home stay

## Abstract

**Background:**

Older people with diabetes who live at home and receive home care services (HCS) are vulnerable, which may result in a need for more care than the HCS can provide. In this study we aimed to explore associations between pharmacologically treated diabetes and the risk of short-term and long-term nursing home stays (NHS) among older people receiving HCS.

**Methods:**

This nationwide registry study included older people ≥ 65 years receiving HCS, as registered in the Norwegian Information System for the Nursing and Care Sector (IPLOS) (2010–2014). Data from IPLOS were merged with data from the Norwegian Prescription Database and the Norwegian Patient Registry. Pharmacologically treated diabetes (hereafter referred to as diabetes) was defined based on prescriptions of glucose-lowering drugs (GLD) (≥ 1 prescription in the current or previous year). Persons not prescribed GLD were defined as not having diabetes. Based on Anatomical Therapeutic Chemical (ATC) codes we identified the following subgroups: persons without diabetes, persons using “non-insulin GLD only”, “insulin and non-insulin GLD” and “insulin only”. An NHS was defined as at least one stay during a given calendar year, where a short-term NHS is temporary, and a long-term NHS is permanent. Log-binomial regression was used to test for differences in NHS and results are reported as risk ratios (RR) with 95% CIs.

**Results:**

Both insulin-treated subgroups had a higher risk of a short-term NHS (“insulin only” users RR 1.06 (CI 1.03–1.09) and “insulin and non-insulin GLD” users RR 1.04 (CI 1.02–1.06)) compared to those without diabetes. In general, persons with diabetes had a lower risk of a long-term NHS than those without diabetes (RR 0.92 (CI 0.89–0.94)). The subgroup using “insulin and non-insulin GLD” had the lowest risk of a long-term NHS (RR 0.86 (CI 0.81–0.91)).

**Conclusion:**

Despite a lower risk of a long-term NHS among older people with diabetes, we found an increased risk of a short-term NHS among persons with insulin-treated diabetes who live at home and receive HCS. This calls for attention when planning health care, in order to provide coordinated and individualized care to prevent short-term NHS’s.

**Supplementary Information:**

The online version contains supplementary material available at 10.1186/s12877-024-05403-5.

## Background

Despite the widespread international attention to increasing diabetes prevalence [1], care needs for older people with diabetes receiving home care services (HCS) are scarcely studied. Older people with diabetes are a heterogeneous group with higher rates of multimorbidity and functional disability than other older adults, and also a higher risk of common geriatric syndromes [2–4]. Moreover, deteriorating cognitive and physical ability may challenge their ability to maintain a stable blood glucose and thus call for more support from significant others or healthcare professionals [2].

Furthermore, multimorbidity is found to be associated with higher utilization of health care services among community-dwelling older people in studies from Switzerland and Canada [5, 6]. These studies highlight the need for strategies to improve coordination and better planning of health care services to persons with multimorbidity. Previous research suggests that diabetes is associated with an increased risk of being admitted to a nursing home [7, 8]. Diabetes is also found to be a risk factor for hospitalization and extended hospital stay among HCS recipients [9].

In Norway, individuals who meet specific disability criteria have the option to apply for HCS or a nursing home stay, that are financially supported by Norwegian health authorities [10, 11]. Preferably, persons in need of healthcare should remain at home as long as possible before moving to a nursing home, as the healthcare policy is based on treatment at the lowest effective service level [10–12]. In the period 2002–2017, there was an increase of approximately 20% in the total population receiving HCS (from approximately 160,000–190,000 persons) [13]. The municipalities organize and provide long-term care, and set the eligibility criteria for both HCS and nursing home stays based on national legislation. In addition, general practitioners give recommendations of appropriate level of care for persons applying for long-term care. Short-term nursing home stays are temporary, and often necessary following a hospital stay, as rehabilitation or as respite care for next kin [13, 14]. The length of a short-term stay can vary from a few days up to several weeks, depending on the need. Long-term nursing home stays are permanent and are intended for people in need of a high degree of medical care and nursing services [13]. This is the highest level of care, intended for those who are unable to remain living in their own homes [11].

The vulnerability of older people with diabetes who live at home and receive HCS may result in a need for more care than the HCS is able to provide. Short-term and long-term nursing home stays can therefore be considered as indicators of unmet care needs. Understanding the risk of nursing home stays among people with and without diabetes can help municipalities to assess the care needs of older people with diabetes. Therefore, this study aims to explore the associations between pharmacologically treated diabetes and the risk of short-term and long-term nursing home stays among older people receiving HCS. In addition, we explored differences in nursing home stays between diabetes treatment subgroups and whether the associations with nursing home stays varied depending on living alone or not.

## Methods

### Study design and setting

The study is a nationwide registry-based observational study, including people 65 years and older, receiving HCS as registered in the Norwegian Information System for the Nursing and Care Sector (IPLOS). We included data from 1 January 2009 to 31 December 2014 from IPLOS and merged these with data from the Norwegian Prescription Database (NorPD) and the Norwegian Patient Registry (NPR) for the same time period.

From IPLOS we utilized information on the amount of received time of HCS, nursing home stays (short-term and long-term) and living situation (alone/ not alone). As persons receiving only a limited amount of HCS may not be representative of the general HCS population, persons receiving < 14 h or < 14 separate days of HCS in a given calendar year were excluded from the study population. Data from IPLOS were merged with data on dispensed prescriptions from the NorPD and discharge diagnoses from the NPR. Additional information such as year of birth and sex were retrieved from Statistics Norway (SSB). In order to have at least one year of prescription data and hospitalization data before start of the study period, we defined the study period to be 2010–2014.

### Definition of diabetes

Data on dispensed prescriptions from NorPD were utilized to identify persons with pharmacologically treated diabetes, and we retrieved information on prescriptions for glucose lowering drugs (GLD) with Anatomical Therapeutic Chemical (ATC) code A10A (insulins and analogues), referred to as insulin, and ATC code A10B (blood glucose lowering drugs, excl. insulins), referred to as non-insulin GLD [15].

Individuals with ≥ 1 prescription of GLD in the current or previous year were defined as having pharmacologically treated diabetes. Subsequently, four subgroups within the study population were identified: (1) persons not prescribed GLD (current or previous year), defined as not having pharmacologically treated diabetes, and the diabetes treatment groups; (2) persons prescribed non-insulin GLD only (≥ 1 prescription of non-insulin GLD current or previous year and no prescription of insulin the current and previous year), (3) persons prescribed both insulin and non-insulin GLD (≥ 1 prescription with insulin and ≥ 1 prescription of non-insulin GLD during the same year the current or previous year) and (4) persons prescribed insulin only (≥ 1 prescription with insulin current or previous year and no prescription of non-insulin GLD the current and previous year). Each persons’ diabetes status and treatment group were updated yearly from 2010 to 2014. For language simplicity, we use the term diabetes to describe pharmacologically treated diabetes throughout the paper. We do not have information from NorPD on persons diagnosed with diabetes who do not receive pharmacological treatment, and they are therefore included in the group referred to as “Not diabetes”.

### Outcome measures and covariates

The outcomes were short-term and long-term nursing home stays. Short-term nursing home stays were defined as at least one short-term stay in a nursing home during a given calendar year. A person was defined as having a long-term nursing home stay if he/ she moved permanently to a nursing home during a given calendar year.

Multimorbidity, used as a covariate, was assessed by the Charlson Comorbidity Index (CCI), using the updated Charlson weights as described by Quan et al. [16] based on discharge diagnoses from hospitalizations, using the 10th revision of International Classification of Diseases (ICD-10) code. An individual score was calculated per January 1st each year based on primary and secondary diagnoses reported during hospitalizations the previous year, by using ICD-10 codes. Subsequently, persons who did not have a hospital admission during the previous year had a CCI of 0 per January 1st for a given year. The CCI was categorized into three score categories: 0, 1–2 and ≥ 3. Other measured covariates were age, sex, living situation (living alone or not) and calendar year of observation.

### Statistical analysis

Data were organized in long format with one observation per person per calendar year (2010–2014) with yearly updates of age, living situation (alone/ not alone), diabetes status, treatment subgroup, CCI and binary variables for short-term and long-term nursing home stays. T –tests, chi-square tests and median tests were used to investigate differences in characteristics of the study population between those with and without diabetes within one calendar year (data from 2014). Further, chi-square tests and linear regression were used to test for differences in characteristics between the different treatment subgroups. Age above 90 was truncated to 90 because of regulations for protection of personal data. Log-binominal regression was used to test for treatment subgroup differences in risk of nursing home stays. Exponentiated regression coefficients are presented as risk ratios (RR) along with 95% confidence intervals (CI). When testing for differences in risk of nursing home stays, regression models were estimated with and without adjustment for age, sex, calendar year and multimorbidity (measured by categorized CCI). Age and calendar year were treated as continuous variables and CCI as a categorical variable. As prevalence of diabetes and access to short and long-term nursing homes stays vary over time, we adjusted for calendar year in order to compare diabetes subgroups the same calendar year. In additional analysis we also excluded those who died. Regression models testing for differences in risk of nursing home stays were in addition preformed stratified by living situation (alone/ not alone). Additional analyses on risk of short-term nursing home stays stratified by hospital admission (yes/ no) were adjusted for age, sex, and calendar year. Clustered robust standard errors were used in all regression models to account for the possibility that the same individual could be included in the analysis sample for more than one calendar year. Significance level was defined as *p* < 0.05. All analyses were performed in STATA version 17.

## Results

### Characteristics

The total study population varied from 117,673 in 2010 to 125,593 persons in 2014. Characteristics of the study population in 2014 are presented in Table 1. Among the 15.7% who had diabetes, mean age was lower, a larger proportion were men, a smaller proportion was living alone, and they received more hours of HCS, compared to those without diabetes. The proportion of persons who had a CCI 1–2 and ≥ 3, was higher among those with diabetes compared to persons without diabetes.

**Table 1:** Characteristics of the study population (*N* = 125 593) in 2014

Variable			Not DM^a^	DM^a^	*p* ^b^	Subgroups of DM^a^			
						Non-insulin GLD^c^ only	Insulin and non-insulin GLD^c^	Insulin only	*p* ^b^
N (% of total population)			105,841 (84.3)	19,752 (15.7)		11,192 (8.9)	4,598 (3.7)	3,962 (3.2)	
Female, n (%)			69,707 (65.9)	10,969 (55.5)	< 0.001	6,430 (57.5)	2,457 (53.4)	2,082 (52.6)	< 0.001
Age, mean (SD)			82.1 (7.4)	79.9 (7.4)	< 0.001	80.6 (7.3)	78.4 (7.3)	79.7 (7.5)	< 0.001
Age group n (%)					< 0.001^d^				< 0.001^d^
		65–74	19,906 (18.8)	5,201 (26.3)		2,618 (23.4)	1,496 (32.5)	1,087 (27.4)	
		75–84	35,966 (34.0)	8,133 (41.2)		4,543 (40.6)	1,995 (43.4)	1,595 (40.3)	
		85–89	26,239 (24.8)	4,015 (20.3)		2,459 (22.0)	775 (16.9)	781 (19.7)	
		90+	23,730 (22.4)	2,403 (12.2)		1,572 (14.1)	332 (7.2)	499 (12.6)	
Living alone^e^, n (%)			62,664 (63.6)	10,625 (57.5)	< 0.001	6,267 (60.0)	2,357 (55.0)	2,001 (53.1)	< 0.001
Hours of HCS^f^, median (IQR)			52 (14–160)	62 (18–187)	< 0.001	52 (14–159)	78 (22–208)	91 (26–236)	< 0.001
Charlson comorbidity index, n (%)					< 0.001^d^				< 0.001^d^
	0		86,296 (81.5)	14,622 (74.0)		9,032 (80.7)	3,078 (66.9)	2,512 (63.4)	
	1–2		14,199 (13.4)	3,497 (17.7)		1,540 (13.8)	1,019 (22.2)	938 (23.7)	
	3–14		5,346 (5.1)	1,633 (8.3)		620 (5.5)	501 (10.9)	512 (12.9)	

^a^ DM = Diabetes mellitus, defined as a person registered in the The Norwegian Prescription Database (NorPD) with at least one prescription of Insulins and analogues (A10A) or Blood glucose lowering drugs, excl. insulin (A10B) in the current or previous year

^b^ T –tests, chi-square tests and median test are used to investigate the difference between those with and without pharmacologically treated diabetes. Chi-square tests and linear regression with diabetes subgroup as a categorical independent variable are used to test for difference between diabetes subgroups

^c^ GLD = Glucose lowering drugs

^d^ p-value reflect test of overall difference

^e^ Missing: *n* = 8,616 in 2014

^f^ HCS = Home care services

### Nursing home stays

**Fig. 1:**
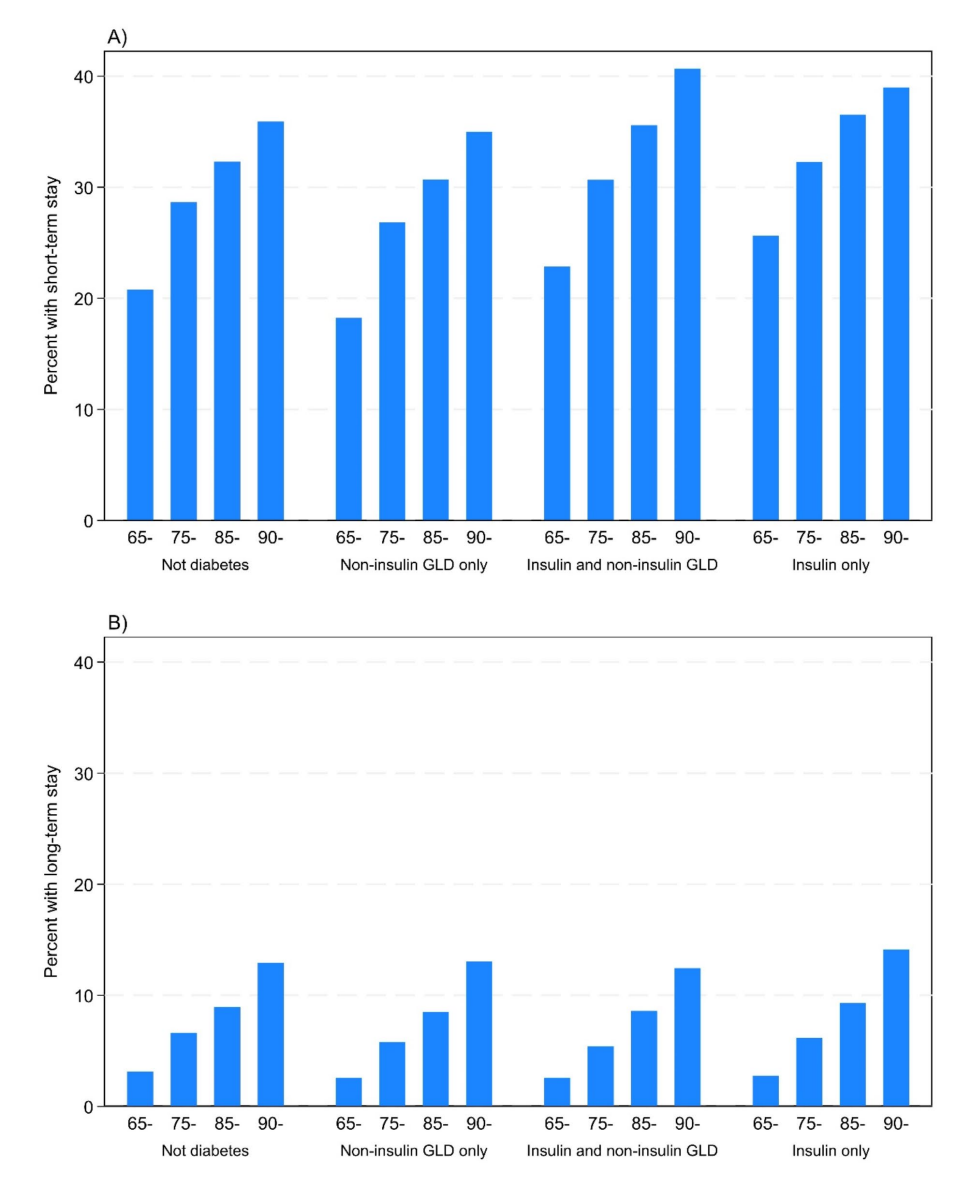
Prevalence of **A**) Short-term NHS^a^ and **B**) Long-term NHS^a^, by diabetes subgroups and age group. ^a^ NHS = Nursing home stay (at least one in a given calendar year) in the period 2010–2014

In general, when combining all groups with diabetes there was a lower proportion with nursing home stays than among those without diabetes (unadjusted counts and percentages shown in Table 2). Persons using “insulin only” had the highest occurrence of at least one short-term nursing home stay in the period 2010–2014, while persons without diabetes had the highest occurrence of long-term nursing home stays (Table 2). Prevalence of short-term and long-term nursing home stays in the different diabetes subgroups and age groups are shown in Fig. 1. Both in people with and without diabetes, persons not living alone had a higher proportion with nursing home stays than those living alone (Table 3).

**Table 2:** Differences in risk of short-term and long-term nursing home stays (2010–2014), by diabetes status

	*N* (%)	Model 1 RR (CI)	Model 2 RR (CI)	Model 3 RR (CI)
	**Short-term NHS** ^**a**^	**Relative difference in risk of a short-term NHS** ^**a**^		
Not DM^b^	153,129 (29.8)	1 (Ref)	1 (Ref)	1 (Ref)
DM^b^	26,976 (28.6)	0.96 (0.95–0.97)	1.01 (0.99–1.02)	0.98 (0.97-1.00)
Subgroups of DM^b^				
Not DM^b^	153,129 (29.8)	1 (Ref)	1 (Ref)	1 (Ref)
Non-insulin GLD^c^ only	14,839 (27.0)	0.91 (0.89–0.92)	0.94 (0.92–0.95)	0.93 (0.92–0.95)
Insulin and non-insulin GLD^c^	6,226 (29.8)	1.00 (0. 98-1.03)	1.09 (1.06–1.11)	1.04 (1.02–1.06)
Insulin only	5,911 (32.2)	1.08 (1.05–1.11)	1.13 (1.10–1.16)	1.06 (1.03–1.09)
	**Long-term NHS** ^**a**^	**Relative difference in risk of a long-term NHS** ^**a**^		
Not DM^b^	40,967 (8.0)	1 (Ref)	1 (Ref)	1 (Ref)
DM^b^	6,127 (6.5)	0.82 (0.80–0.84)	0.93 (0.91–0.96)	0.92 (0.89–0.94)
Subgroups of DM^b^				
Not DM^b^	40,967 (8.0)	1 (Ref)	1 (Ref)	1 (Ref)
Non-insulin GLD^c^ only	3,702 (6.7)	0.85 (0.82–0.87)	0.93 (0.90–0.96)	0.92 (0.89–0.95)
Insulin and non-insulin GLD^c^	1,169 (5.6)	0.70 (0.67–0.74)	0.89 (0.84–0.94)	0.86 (0.81–0.91)
Insulin only	1,256 (6.8)	0.86 (0.81–0.90)	0.99 (0.94–1.05)	0.95 (0.90-1.00)

* Model 1: Unadjusted, Model 2: Adjusted for age, sex and calendar year, Model 3: Adjusted for age, sex, calendar year and multimorbidity (measured by categorized Charlson comorbidity index score). ^a^ NHS = Nursing home stay (at least one) ^b^ DM = Diabetes mellitus, defined as a person registered in the The Norwegian Prescription Database (NorPD) with at least one prescription of Insulins and analogues (A10A) or Blood glucose lowering drugs, excl. insulin (A10B) in the current or previous year. ^c^ GLD = Glucose lowering drugs

**Table 3:** Differences in risk of short-term and long-term nursing home stays (2010–2014), stratified by living situation

	Short-term NHS^a^		Long-term NHS^a^	
	*N* (%)	RR (CI)	*N* (%)	RR (CI)
**Living alone**				
Not DM^b^	87,166 (28.8)	1 (Ref)	19,735 (6.5)	1 (Ref)
DM^b^	14,091 (27.8)	0.99 (0.97–1.01)	2,839 (5.6)	0.95 (0.92–0.99)
Subgroups of DM^b^				
Not DM^b^	87,166 (28.8)	1 (Ref)		1 (Ref)
Non-insulin GLD^c^ only	7,999 (26.0)	0.94 (0.92–0.96)	1,709 (5.6)	0.93 (0.89–0.98)
Insulin and non-insulin GLD^c^	3,122 (29.5)	1.07 (1.03–1.10)	530 (5.0)	0.93 (0.85–1.01)
Insulin only	2,970 (31.7)	1.08 (1.04–1.11)	600 (6.4)	1.05 (0.97–1.14)
**Not living alone**				
Not DM^b^	57,005 (33.7)	1 (Ref)	20,163 (11.9)	1 (Ref)
DM^b^	11,418 (31.5)	0.96 (0.94–0.98)	3,131 (8.6)	0.84 (0.81–0.87)
Subgroups of DM^b^				
Not DM^b^	57,005 (33.7)	1 (Ref)		1 (Ref)
Non-insulin GLD^c^ only	5,999 (30.1)	0.92 (0.90–0.94)	1,894 (9.5)	0.88 (0.84–0.92)
Insulin and non-insulin GLD^c^	2,762 (32.0)	0.99 (0.96–1.03)	612 (7.1)	0.76 (0.71–0.83)
Insulin only	2,657 (34.3)	1.02 (0.98–1.05)	625 (8.1)	0.81 (0.75–0.87)

* Adjusted for age, sex, calendar year and multimorbidity (measured by categorized Charlson comorbidity index score). Missing living situation 2010–2014: *n* = 49,795. ^a^ NHS = Nursing home stay (at least one) ^b^ DM = Diabetes mellitus, defined as a person registered in the The Norwegian Prescription Database (NorPD) with at least one prescription of Insulins and analogues (A10A) or Blood glucose lowering drugs, excl. insulin (A10B) in the current or previous year. ^c^ GLD = Glucose lowering drugs

### Short-term nursing home stays

The risk of a short-term nursing home stay was higher among persons using insulin (both “insulin only” (RR 1.06 (CI 1.03–1.09)) and “insulin and non-insulin GLD” (RR 1.04 (CI 1.02–1.06))), compared to persons not having diabetes (Table 2, model 2 and 3). Persons using “non-insulin GLD only” had lower risk (RR 0.93 (CI 0.92–0.95)) of a short-term nursing home stay than persons without diabetes (Table 2).

Stratified by living situation, persons living alone using “insulin only” or “insulin and non-insulin GLD” had higher risk of a short-term nursing home stay (RR 1.08 (CI 1.04–1.11) and RR 1.07 (CI 1.03–1.10), respectively), compared to those without diabetes. Whether they were living alone or not, persons using “non-insulin GLD only” had a lower risk of a short-term nursing home stay than persons without diabetes (Table 3).

Results from additional analyses showed that persons who had a hospital admission (yes/ no) during the study period had a threefold higher risk of having a short-term nursing home stay the same year (RR 3.08 (CI 3.04–3.11), not shown in tables).

### Long-term nursing home stays

Persons with diabetes had a lower risk of long-term nursing home stays than persons without diabetes (RR 0.92 (CI 0.89–0.94)) (Table 2 and Additional file 1: Supplementary Table 1 (model with RR estimates for all covariates)). When stratified by subgroups, those using “insulin only” did not differ significantly from those without diabetes (adjusted regression models). The subgroup using “insulin and non-insulin GLD” had the lowest risk of a long-term nursing home stay (RR 0.86 (CI 0.81–0.91)) (Table 2). This also applied if those who died during the study period were excluded from the analysis (“insulin and non-insulin GLD” users RR 0.82 (CI 0.77–0.88), not shown in tables).

Among persons not living alone, persons with diabetes had lower risk of long-term nursing home stays compared to other recipients of HCS (Table 3 and Additional file 1: Supplementary Table 2 (model with RR estimates for all covariates)). When living alone, only the subgroup using “non-insulin GLD only” had significant lower risk of a long-term nursing home stay than persons without diabetes (RR 0.93 (CI 0.89–0.98)) (Table 3).

## Discussion

We found that older people with diabetes had a lower risk of long-term nursing home stays compared to other recipients of HCS. However, the risk varied substantially between the diabetes treatment groups “insulin only”, “insulin and non-insulin GLD” and “non-insulin GLD only”. Furthermore, the diabetes subgroups using insulin (“insulin only” or “insulin and non-insulin GLD”) had higher risk of a short-term nursing home stay compared to persons without diabetes.

Overall, persons with diabetes had a lower risk of long-term nursing home stays compared to persons without diabetes in HCS, except for those in the subgroup “insulin only”. This was a surprising finding - as there was more multimorbidity among persons with diabetes. One might assume that persons with diabetes received a more individualized care, tailored to their needs, since they received significantly more hours of HCS. Then again, these persons were able to live longer in their own home. On the other hand, previous studies have found that among community-dwelling older people with diabetes, conditions regarded as non-diabetes related were important drivers of the health care use [17, 18]. A study conducted by Griffith et al. [6], which compared community-dwelling older adults with diabetes, dementia, and stroke, found that the use of health services increased with the number of conditions. However, the index condition was not necessarily the main driver of this increased use. Unfortunately, we do not have information on the primary reason for receiving HCS in the present study. Thus, it might be that those without diabetes have other conditions which require a long-term nursing home stay, such as dementia or physical disabilities. Cognitive impairment, activities of daily living dependencies and prior nursing home admission were found to be strong predictors of nursing home admission in a meta-analysis from the U.S [19]. Still, taken into account that people using “Insulin and non-insulin GLD”, are likely to have a more advanced type 2 diabetes condition [2], it is interesting that they have the lowest risk of a long-term nursing home stay in this HCS population. The subgroup using “Insulin and non-insulin GLD” also had the lowest mean age in our study population. One possible explanation could be that increased mortality contributed to the decreased risk of a long-term nursing home stay for those with a more complex and demanding comorbid condition. However, since the lower risk of long-term nursing home stays was still present after excluding those who died, this is not likely.

In this population of HCS recipients, we found that older people with insulin-treated diabetes had a higher risk of short-term nursing home stays compared to persons without diabetes. Given the higher level of multimorbidity among persons treated with insulin, the increased risk of a short-term nursing home stay may reflect periodic requests for advanced individualized monitoring. To what degree the increased risk of a short-term nursing home stay is related to the complexity of insulin-treatment, needs further investigation. In a recent Norwegian study, an unacceptably high number of unknown hypoglycaemic episodes among older people with diabetes receiving home care was identified [20].

Glucose-monitoring and insulin treatment can be challenging for both the patient and health care workers indicating that older persons with diabetes might need a more intensified and structured monitoring of blood glucose levels throughout the day to avoid a short-term nursing home stay. Within our study population, we have previously reported that insulin-users have higher risk for hospitalization [9]. Previous research studies have found a lack of documentation related to diabetes care in HCS, which can compromise the patient safety [21, 22]. In a review article, Haltbakk et al. [23] revealed that persons with diabetes in HCS are at risk of adverse advents due to their reduced ability to self-manage the condition, adverse medication effects, the family’s ability to take responsibility for management of the condition, or suboptimal approaches to diabetes care in HCS. Considering these findings, our results on short-term nursing home stays among insulin users, might suggest that it may be challenging to provide accurate care for this patient group at home.

Overall, in our study population persons with a cohabitant had a higher risk of both short-term and long-term nursing home stays. Respite care for informal caregivers may be a reason for this [24], or perhaps a resourceful cohabitant is better able to advocate the recipients needs of a nursing home stay [11]. Further, we found that persons with diabetes using insulin and living alone had higher risk of a short-term nursing home stay, compared to persons without diabetes who were living alone. Possibly, the complexity of insulin treatment is an explanatory factor. This difference is not seen among those living with others, so informal care may prevent short-term nursing home stays among insulin-users. Furthermore, insulin-users who were living with someone had a lower risk of long-term nursing home stays than those living alone, when compared to the reference groups. Based on these results, it seems likely that “living with someone” is more preventive of nursing home stays for persons with insulin-treated diabetes, compared to other recipients of HCS. Further research is needed to determine not only triggering, but also protective factors for nursing home stays among recipients of HCS.

The use of nationwide registry data is a major strength of this study, especially with regards to the lack of previous research in home care settings. It is also a strength that we have valid information on ATC prescriptions of insulin (registry data), which makes it possible to explore differences between diabetes subgroups. However, the absence of more specific information on diabetes diagnosis is a limitation. Such information would have allowed us to more accurately differentiate type 1 and type 2 diabetes. Additionally, this information could have identified individuals who had been diagnosed with diabetes but were not receiving pharmaceutical treatment. Another limitation is that we do not have information from the IPLOS registry on why people receive HCS, we do not know if diabetes is the primary reason for receiving HCS for those with diabetes, nor do we know the primary reason why those without diabetes receive HCS. We are using information from yearly updates on diabetes status and nursing home stays because personal data protection regulations prevented us from using exact dates. Because of this, there is some uncertainty related to which event comes first. However, among persons receiving HCS more than one year in our data, 96% of those registered with diabetes one year also had diabetes the previous year. Thus, we believe that this is not a major source of bias when studying associations between diabetes and short-term stays. If someone received a diagnosis of diabetes after moving to a nursing home permanently, this will not be registered as diabetes in our dataset. We included “diabetes with complications” in the CCI and this might therefore contribute to a higher level of multimorbidity among those with diabetes, given they were hospitalized in the previous year. However, as the comparison group without diabetes in this homecare setting is also expected to have diagnoses that are included in the CCI, we did not exclude diabetes from the score. As we aimed to estimate multimorbidity and not morbidity co-morbid to a main condition, we believe it is most correct to include diabetes in the multimorbidity score. Another limitation related to the measurement of multimorbidity is that by defining the CCI based on diagnosis reported during hospitalization, only the most severe multimorbidity which required hospitalization the previous year is counted, while less serious and more stable conditions treated in primary care or outpatient clinics are not included. Finally, as merging of our registry files was a complex process, the most recent data is from 2014. However, the results are still relevant and important for further strategy planning. Despite these limitations, this population-based study in home care should provide a reasonable representation of the risk of short-term and long-term nursing home stays among older people with diabetes compared to other recipients of HCS in Norway.

## Conclusions

Older Norwegians in HCS with diabetes using insulin treatment have an increased risk of a short-term nursing home stay, while those with diabetes overall have a lower risk of a long-term nursing home stay compared to other persons in HCS. Our results emphasize that older people with diabetes are a heterogeneous group, underscoring the importance of coordinated and individualized HCS. More short-term nursing home stays among insulin-users who live at home and receive HCS may suggest unmet care needs. However, further research is necessary to determine the causes of these short-term stays and whether some can be prevented.

## Electronic supplementary material

Below is the link to the electronic supplementary material.

Supplementary Material 1

## Data Availability

The datasets generated and/or analysed during the current study are not publicly available due to data protection regulations. Personal data protection legislation and the approval from the Regional Ethical Committee prohibits data sharing for the purpose of reproducing the results. Researchers can apply for ethical approval and obtain data from the registry holders (service@helsedata.no).

## Abbreviations

ATC: Anatomical Therapeutic Chemical
CCI: Charlson Comorbidity Index
DM: Diabetes mellitus
GLD: Glucose lowering drugs
HCS: Home care services
ICD-10: The 10th revision of International Classification of Diseases codes
IPLOS: The Norwegian Information System for the Nursing and Care Sector
NHS: Nursing home stay
NorPD: The Norwegian Prescription Database
RR: Risk ratios
SSB: Statistics Norway
