# Supplementary material for: Associations between diabetes and risk of short-term and long-term nursing home stays among older people receiving home care services: A nationwide registry study
Source: BMC Geriatr. 2024 Oct 9;24:814. doi: 10.1186/s12877-024-05403-5 (PMC11462714; doi:10.1186/s12877-024-05403-5)
Supplement: Supplementary file 1 — Supplementary Material 1 [file 12877_2024_5403_MOESM1_ESM.docx]

**Additional file 1**

| **Variables** | | **Model 1**  **RR (CI)** | **Model 2**  **RR (CI)** | **Model 3**  **RR (CI)** |
| --- | --- | --- | --- | --- |
|  | | **Relative difference in risk of a short-term NHS^a^** | | |
| Not DM^b^ | | 1 (Ref) | 1 (Ref) | 1 (Ref) |
| DM^b^ | | 0.96 (0.95-0.97) | 1.01 (0.99-1.02) | 0.98 (0.97-1.00) |
| Age | |  | 1.03 (1.03-1.03) | 1.03 (1.03-1.03) |
| Sex | |  |  |  |
|  | Men |  | 1 (Ref) | 1 (Ref) |
|  | Women |  | 0.90 (0.89-0.91) | 0.93 (0.92-0.94) |
| Calendar year | |  | 1.04 (1.03-1.04) | 1.04 (1.03-1.04) |
| Charlson Comorbidity Index | |  |  |  |
|  | 0 |  |  | 1 (Ref) |
|  | 1-2 |  |  | 1.34 (1.33-1.35) |
|  | 3-14 |  |  | 1.60 (1.58-1.62) |
|  | |  | | |
|  | | **Relative difference in risk of a long-term NHS^a^** | | |
| Not DM^b^ | | 1 (Ref) | 1 (Ref) | 1 (Ref) |
| DM^b^ | | 0.82 (0.80-0.84) | 0.93 (0.91-0.96) | 0.92 (0.89-0.94) |
| Age | |  | 1.07 (1.07-1.07) | 1.07 (1.07-1.07) |
| Sex | |  |  |  |
|  | Men |  | 1 (Ref) | 1 (Ref) |
|  | Women |  | 0.94 (0.92-0.96) | 0.96 (0.94-0.98) |
| Calendar year | |  | 1.00 (0.99-1.00) | 1.00 (0.99-1.00) |
| Charlson Comorbidity Index | |  |  |  |
|  | 0 |  |  | 1 (Ref) |
|  | 1-2 |  |  | 1.31 (1.28-1.34) |
|  | 3-14 |  |  | 1.39 (1.34-1.44) |

**Supplementary Table 1: Differences in risk of short-term and long-term nursing home stays (2010-2014) between persons with and without pharmacologically treated diabetes in home care services in Norway.**

* Model 1: Unadjusted, Model 2: Adjusted for age, sex and calendar year, Model 3: Adjusted for age, sex, calendar year and multimorbidity (measured by categorized Charlson comorbidity index score). ^a^ NHS = Nursing home stay (at least one) ^b^ DM = Diabetes mellitus, defined as a person registered in the The Norwegian Prescription Database (NorPD) with at least one prescription of Insulins and analogues (A10A) or Blood glucose lowering drugs, excl. insulin (A10B) in the current or previous year.

**Supplementary Table 2: Differences in risk of short-term and long-term nursing home stays (2010-2014) between persons with and without pharmacologically treated diabetes in home care services in Norway, stratified by living situation.**

|  | | **Short-term NHS^a^** | **Long-term NHS^a^** |
| --- | --- | --- | --- |
| **Variables** | | **RR (CI)** | **RR (CI)** |
| **Living alone** | |  |  |
| Not DM^b^ | | 1 (Ref) | 1 (Ref) |
| DM^b^ | | 0.99 (0.97-1.01) | 0.95 (0.92-0.99) |
| Age | | 1.03 (1.03-1.03) | 1.07 (1.07-1.08) |
| Sex | |  |  |
|  | Men | 1 (Ref) | 1 (Ref) |
|  | Women | 0.96 (0.94-0.97) | 0.90 (0.87-0.92) |
| Calendar year | | 1.04 (1.03-1.04) | 1.00 (0.99-1.01) |
| Charlson Comorbidity Index | |  |  |
|  | 0 | 1 (Ref) | 1 (Ref) |
|  | 1-2 | 1.37 (1.35-1.39) | 1.35 (1.30-1.40) |
|  | 3-14 | 1.69 (1.66-1.72) | 1.56 (1.48-1.64) |
|  | |  |  |
| **Not living alone** | |  |  |
| Not DM^b^ | | 1 (Ref) | 1 (Ref) |
| DM^b^ | | 0.96 (0.94-0.98) | 0.84 (0.81-0.87) |
| Age | | 1.03 (1.03-1.03) | 1.08 (1.08-1-08) |
| Sex | |  |  |
|  | Men | 1 (Ref) | 1 (Ref) |
|  | Women | 0.95 (0.94-0.97) | 1.32 (1.29-1.35) |
| Calendar year | | 1.03 (1.02-1.03) | 0.99 (0.98-1.00) |
| Charlson Comorbidity Index | |  |  |
|  | 0 | 1 (Ref) | 1 (Ref) |
|  | 1-2 | 1.27 (1.25-1.29) | 1.16 (1.12-1.20) |
|  | 3-14 | 1.45 (1.42-1.48) | - 1. (1.06-1.17) |

*Adjusted for age, sex, calendar year and multimorbidity (measured by categorized Charlson comorbidity index score). Missing living situation 2010-2014: n = 49,795. ^a^ NHS = Nursing home stay (at least one) ^b^ DM = Diabetes mellitus, defined as a person registered in the The Norwegian Prescription Database (NorPD) with at least one prescription of Insulins and analogues (A10A) or Blood glucose lowering drugs, excl. insulin (A10B) in the current or previous year.
